# Hybrid YOLOv3 and ReID intelligent identification statistical model for people flow in public places

**DOI:** 10.1038/s41598-024-64905-9

**Published:** 2024-06-25

**Authors:** Yao Zheng

**Affiliations:** TUM School of Computation, Information and Technology, Technical University Munich, 85748 Munich, Germany

**Keywords:** People flow identification, Pedestrians, Public places, YOLOv3, ReID, DeepSORT, Engineering, Materials science, Mathematics and computing

## Abstract

The statistical model for automatic flow recognition is significant for public place management. However, the current model suffers from insufficient statistical accuracy and low lightweight. Therefore, in this study, the structure of the lightweight object detection model "You Only Live Once v3" is optimized, and the "Deep Simple Online Real-Time Tracking" algorithm with the "Person Re-Identification" module is designed, so as to construct a statistical model for people flow recognition. The results showed that the median PersonAP of the designed model was 94.2%, the total detection time was 216 ms, the Rank-1 and Rank-10 were 87.2% and 98.6%, respectively, and the maximum occupied memory of the whole test set was 2.57 MB, which was better than all comparison models. The results indicate that the intelligent identification statistical model for public crowd flow obtained through this design and training has higher statistical accuracy, less computational resource consumption, and faster computing speed. This has certain application space in the management and guidance of crowd flow in public places.

## Introduction

Against the backdrop of increasing emphasis on intelligent monitoring and scene analysis, pedestrian flow statistics, as a key technology, plays a crucial role in the public transport sector, retail, public safety, and urban planning^1,2^. Advances in object detection and object tracking technology provide technical support for accurate crowd statistics^3^. Object detection is capable of locating and identifying pedestrians in images, while object tracking is responsible for maintaining the identity consistency of the target in the video frame sequence^4–6^. Typical object detection algorithms, such as the You Only Look Once (YOLO) series of algorithms, provide a powerful impetus for identifying crowds in public places through their fast and accurate detection capabilities^7^. As the third-generation algorithm of this series, YOLOv3 has become a popular choice due to its excellent real-time performance and high recognition accuracy.

Current technologies show certain limitations when dealing with the flow of people in large-scale public places, especially in terms of statistical accuracy and computational efficiency^8^. Traditional models face many challenges, such as model bloat, high computing power requirements, and slow processing speed^9^. In addition, for public places with complex backgrounds and dense crowds, problems such as real-time tracking accuracy still exist. Therefore, this study proposes a statistical model that combines YOLOv3 and Re-Identification (ReID) for intelligent recognition of human flow, aiming at the shortcomings of the existing models. The network architecture of the YOLOv3 model is adjusted, the lightweight technology is used to reduce the computational cost, and the individual tracking performance of the model is enhanced by introducing the ReID module. The multi-target tracking ability of the model is optimized by the Deep Simple Online Realtime Tracking (DeepSORT) algorithm.

This study includes four parts. The first part is the research background and research motivation in object detection and tracking. The second part describes in detail the design process of the improved YOLO lightweight model for human flow intelligence statistics. The third part shows the comparative test of the proposed model and the existing technology, and analyzes the results to verify the method superiority. The fourth part summarizes the research and looks forward to the future work.

## Related works

The people flow statistics model includes three parts: object detection, target tracking, and target counting. After this type of model detects the required target, it tracks the target to determine whether the target triggers the counting rule to carry out the flow statistics. The problem of target counting is difficult to solve, so the core of the crowd detection model lies in object detection and target tracking.

In terms of object detection, Choi et al. proposed that how to identify the authenticity of news has become a hot research topic due to the frequent occurrence of fake news videos. Most of the existing studies were based on machine learning and deep learning technologies, using domain knowledge and multi-modal data fusion technology to identify the authenticity of video content. This study tried to judge the authenticity of video news by identifying key markers in video images through object detection technology. An object detection model based on YOLOv5 algorithm was constructed. The test results showed a recognition accuracy of 97.3% for the key markers that distinguished 100% between the authenticity of the news, which was significantly higher than that of the comparison detection model^10^. In object detection and behavior recognition, the auto-encoder-based frame prediction method has excellent abnormal behavior detection ability. However, the current method has some problems, such as the detection failure caused by the strong representation ability of the auto-encoder and insufficient ability to extract spatiotemporal information. In this paper, a new network framework combining pseudo-3D convolution and multi-cascading memory mechanism was proposed to effectively solve these problems. Its detection efficiency and superiority were verified on public datasets^11^. In order to improve the data scarcity in supervised visual tasks, self supervised label enhancement technology has emerged, which has shown significant effects in the field of object detection. Traditional self supervised label enhancement methods may overlook necessary supervised information, which may affect classification performance. Therefore, W. Gao et al. proposed a detached self-supervised label enhancement method, which improved the performance of fully supervised image classification by enhancing feature representation. The results showed that it had advantages over current popular data augmentation methods on multiple datasets^12^. Gao et al. proposed a novel pairwise attention network. The network generated different attention evaluators by combining different local features with global features to obtain an attention map that focused on important parts of the image and ignored irrelevant areas. In addition, in order to ensure that images of the same category had a consistent area of interest, PAN also added an attention consistency mechanism. A large number of results on the Iris and Visda-2017 datasets showed that the designed model outperformed the existing advanced methods in average accuracy of object detection^13^. Fang used deep residual learning convolutional network to improve neural network performance degradation with the increase of layers. The ArcFace loss function was combined to optimize the ResNet network to achieve the face detection accuracy. Experimental results showed that the model stably detected and recognized faces even under facial defects and bright light exposure^14^.

In terms of object tracking, Liu et al. proposed an object tracking model for particle images, which adjusted the core network structure of the DeepSORT algorithm and greatly reduced the model parameters. The results showed that the target tracking accuracy on a particle image dataset was 98.6%, which was much higher than the improved DeepSORT algorithm^15^. Jeong et al. discussed a real-time computer vision perception method using deep learning to solve the object tracking uncertainty caused by lighting changes and field of view occlusion, and maintained the real-time processing power of the model. Indoor and outdoor experiments were conducted to evaluate the performance of the design model under the light change, shading, and haze. Comparative experiments showed that this method was superior to many other methods, demonstrating its feasibility for long-term displacement monitoring of full-scale structures^16^.

Related research has made some progress in the human flow statistical models, but there are still significant shortcomings. In the object detection, although deep learning methods such as YOLOv5 and auto-encoder frameworks have achieved high recognition accuracy, they face challenges such as detection failure caused by the strong representation ability of auto-encoders and insufficient spatiotemporal information extraction ability. In terms of target tracking, although the DeepSORT algorithm and its variants provide high tracking accuracy, real-time tracking accuracy and computational efficiency are limited in complex backgrounds and crowded scenes. In response to these shortcomings, this study proposes an intelligent recognition statistical model that combines YOLOv3 and ReID. This model adjusts the network architecture of YOLOv3, adopts lightweight technology to reduce computational cost, and introduces a ReID module to improve personal tracking performance. In addition, optimizing the DeepSORT algorithm enhances the model multi-target tracking ability. These improvement measures effectively address the shortcomings of existing models in terms of statistical accuracy, computational efficiency, and real-time tracking accuracy, enabling the method proposed in this study to more accurately measure crowd flow while maintaining lower computational cost and higher real-time performance, providing strong technical support for crowd management in public places.

## Improved YOLO lightweight model design for intelligent statistics of crowd flow

Crowd statistics can provide data support for the safety management of public places such as bus stops, transportation hubs, and gymnasiums^17–19^. However, the traditional statistical methods based on machine learning algorithms are inefficient and have large statistical errors. Therefore, this research constructs an intelligent statistical model with advanced performance based on YOLOv3 algorithm.

### Lightweight crowd flow detection algorithm based on improved YOLO

YOLOv3, as a single-stage detector, has shown many superior characteristics in the pedestrian detection. Compared with other updated versions of the YOLO series, the YOLOv3 model, as a classic and widely used object detection algorithm, has been fully validated in terms of network structure and performance. The relative simplicity of the YOLOv3 model also makes it easier to modify and optimize to verify the effectiveness of newly proposed methods or strategies. Furthermore, the YOLOv3 has lower computational resource requirements relative to the updated models. In this resource-limited environment, YOLOv3 is more appropriate. Firstly, YOLOv3 fully considers the global nature of the object detection problem. It uses a global convolutional structure to predict the position and class of all objects simultaneously in a forward propagation process^20,21^. Compared with other neural networks and machine learning algorithms, YOLOv3 has a faster detection speed on pedestrian detection tasks, which meets the real-time applications^22^. In addition, YOLOv3 optimizes the objective function design and introduces multi-scale prediction, which can better handle pedestrians of various sizes and shapes, and weaken the detection performance degradation caused by scale changes^9,23^. Meanwhile, compared with the previous version of YOLO, YOLOv3 has added presets for more types of anchor boxes, which can better adapt to pedestrians in various postures, and correspondingly improve the detection accuracy^24^. Therefore, compared with other neural networks and machine learning algorithms, YOLOv3 achieves a good balance between detection speed and accuracy in pedestrian detection tasks, which has significant advantages. Therefore, the YOLOv3 algorithm is selected to build a statistical model for pedestrian flow.

**Figure 1:**
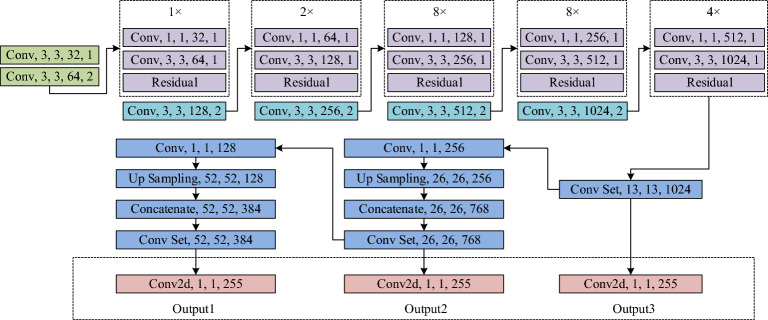
YOLOv3 network structure.

As the third-generation algorithm of YOLOv3, YOLOv3 is different from the previous version in that YOLOv3 has three significant improvements. Firstly, YOLOv3 adopts the concept of ResNet residual network to ensure that the model can converge normally even in extremely deep network structures. Secondly, YOLOv3 implements a pyramid-like network structure, providing multi-scale prediction at three different sizes, especially enhancing the detection performance of small-size targets. Thirdly, in terms of the loss function, YOLOv3 uses binary cross-entropy loss instead of the traditional softmax loss function, which not only improves the target prediction accuracy, but also allows each bounding box to predict multiple targets^25–27^. YOLOv3 feature extraction network Darknet-53 includes many 3 × 3 and 1 × 1 convolutional layers, mainly composed of convolution and residual layers. Its complex depth structure is the reason for the slowdown of YOLOv3 training and detection^28^. Five down-sampling times are carried out in the network, and the features are output in the last three layers, and three scales of YOLO layers are generated after processing in the pyramid feature space^29,30^. Darknet-53 dominates the feature extraction work, and the YOLO layer is responsible for the interaction between the different feature layers. The above improvements make YOLOv3 have stronger human image recognition capabilities, which is one of the reasons for choosing this algorithm to build the model. In Fig. 1, "Conv" is the abbreviation of "Convolutional", and "Conv2d" stands for two-dimensional convolution. The numbers after each layer type character in the figure are filter size, number of neurons, and step size from left to right.

**Figure 2:**
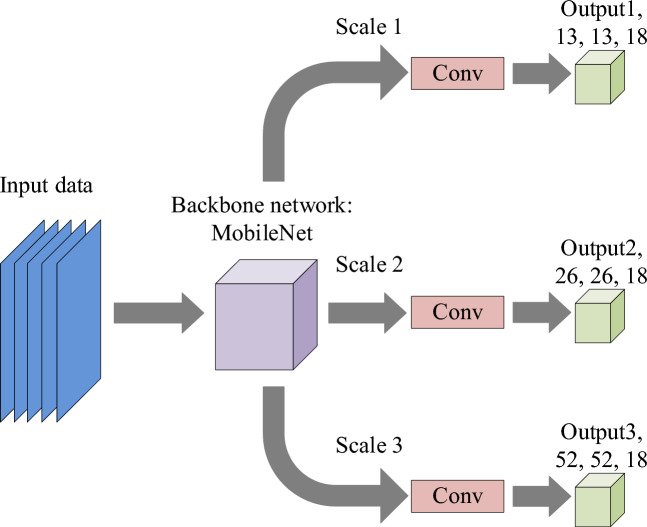
Schematic diagram of I_YOLOv3 algorithm.

YOLOv3 has shown excellent results in the field of pedestrian detection, but it still faces two major challenges. One is the increase in the required storage space due to massive network parameters, which seriously affects its real-time performance in the detection process. The other is that in the changing street background, the pedestrian target size difference and occlusion are different, which is easy to cause missed detection. The main network of YOLOv3, Darknet-53, includes 52 convolutional layers and a fully connected layer, which hardly satisfy real-time requirements based on massive computing and parameters. In contrast, YOLOv3-tiny is a lightweight detection method with less than 9 million parameters and only 33.7 MB storage space, so it has fast training, low video memory requirements, and fast detection. However, its ability to extract complex image features is weak, and its detection performance is poor for light drastic changes, serious occlusion or small targets. In view of this, this chapter is improved based on YOLOv3 and proposes a I_YOLOv3 algorithm. The overall architecture is shown below. The number after the output layer character in Fig. 2 represents the output size.

The first step of the I_YOLOv3 algorithm is network prediction, which uses the I_YOLOv3 network model for forward propagation. The network uses MobileNet as the backbone network and replaces Darknet-53 in YOLOv3 with depth separable convolution, reducing parameters and computational complexity. The second step is to perform output processing by reshaping the tensor output of the network into appropriate dimensions. Each grid cell predicts multiple bounding boxes, including bounding box coordinates, category confidence, and object confidence. The third part is confidence screening, which applies a sigmoid function to the object confidence of each bounding box and sets a threshold. Boundaries with a confidence level higher than the threshold are filtered out as possible detection targets. The fourth step is coordinate inversion. Based on the prediction method of I_YOLOv3, the actual coordinates of the bounding box are calculated by the offset of the bounding box and the prior box information obtained through K-means clustering. The fifth step is to perform Non Maximum Suppression (NMS) and apply NMS to the predicted bounding boxes of the same category, eliminating redundant boxes with high overlap and retaining the bounding box with the highest score. The sixth step outputs the results, draws the filtered bounding boxes on the original image, and labels the predicted categories to complete the pedestrian detection task. The output size O of the I_YOLOv3 algorithm follows Eq. (1).

1 $$O = \left( {I - K + 2P} \right)/S + 1$$

**Figure 3:**
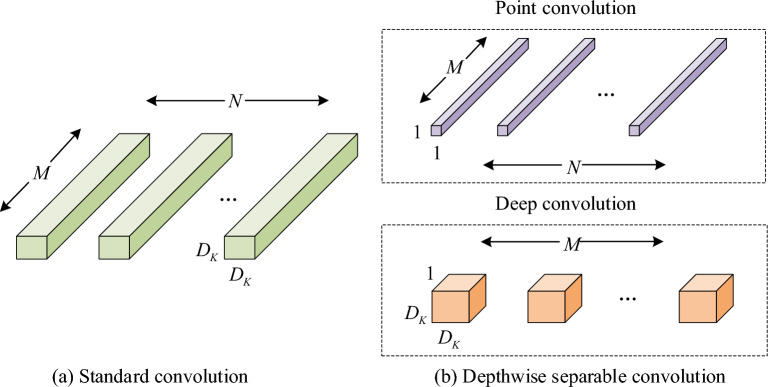
Deep separable convolution and standard convolution structures.

In Eq. (1), $I$ represents the input image size. $K$ represents the convolution kernel size. $P$ represents the number of fills. $S$ represents the convolution step size. In the backbone network, Darknet-53 is replaced by a MobileNet lightweight network using deep separable convolution, because the latter can reduce the parameters and computational complexity of the entire algorithm, which is more suitable for the application scenario of crowd statistics in public places. The deep separable convolution and standard convolution structures are shown in Fig. 3.

Point convolution uses separate convolution kernels to process independent channels. $D_{F}$ is the width and height of the feature map. $M$ and $N$ are the number of deep convolutional layers and point convolutional layers. $D_{K}$ is the width and height of the convolution kernel of the deep convolution and standard convolution. If the size of the output feature map is $D_{F} \times D_{F}$ and the convolution kernel is $K \times K$, the computational amount $S_{1}$ of the standard convolution is shown in Eq. (2).

2 $$S_{1} = D_{K} \times D_{K} \times M \times N \times D_{F} \times D_{F}$$

In contrast, the computational amount $S_{2}$ of depth separable convolution is shown in Eq. (3).

3 $$S_{2} = D_{K} \times D_{K} \times M \times D_{F} \times D_{F} + M \times N \times D_{F} \times D_{F}$$

The two items before and after the plus sign in Eq. (3) are the computational amount of depth convolution and point convolution, respectively. Therefore, the ratio of the computational amount of standard convolution to the deep separable convolution can be described in Eq. (4).

4 $$\frac{{S_{2} }}{{S_{1} }} = \frac{1}{N} + \frac{1}{{D_{k}^{2} }}$$

Equation (4) shows that the computational cost of the depth separable convolution is $\frac{1}{N} + \frac{1}{{D_{k}^{2} }}$ times of the standard convolution. For example, when $N$ is much larger than $K^{2}$, $D_{k}^{{}}$ is 4, and the computational cost of the depth separable convolution is 8/9 lower than that of the traditional convolution. With this approach, MobileNet significantly reduces the amount of computation and parameters, thereby reducing redundant expression.

Before predicting the target box, the anchor boxes are redefined by using K-means clustering in the algorithm to accelerate the model convergence. In this process, the size ratio of the K-means cluster is quite different from the target in the dataset, because the initial candidate box selection does not consider the actual labeling information of the actual pedestrian dataset, which increases the missed detection of small pedestrians. The K-means clustering method optimizes the size and proportion based on the concept of anchor boxes. The clustering distance is measured by calculating the intersection over union ratio (IoU) between the predicted candidate box and the actual bounding box. The distance calculation method for clustering is shown in Eq. (5):

5 $$D\left( {box,center} \right) = 1 - IoU\left( {box,center} \right)$$

In Eq. (5), $center$ is the cluster center and $box$ is the clustering sample.

### Mixed improvement of the flow statistics model based on YOLO and ReID processing

The improved YOLO algorithm designed above is used to detect pedestrian targets. However, in the actual application scenario, the input data contains video, and the detected pedestrians need to be tracked in order to count the flow. Therefore, the pedestrian tracking intelligent algorithm is designed.

DeepSORT is a typical and commonly used object tracking algorithm, which applies Kalman filter in image space to complete target prediction, and uses Hungarian algorithm to correlate inter-frame data one by one. The algorithm measures the correlation using the overlap ratio of the bounding boxes, and shows excellent performance in high frame rate videos. The computational cost of the improved YOLO algorithm is greatly reduced. Due to the shallow network and poor adaptability to the dataset, the ReID module in DeepSORT is improved to obtain Improved DeepSORT (I_DeepSORT). The main improvement of this algorithm is the Convolutional Neural Network (CNN) architecture based on deep cosine metric learning, so as to improve the extraction performance of appearance features. In terms of measurement calculation, the algorithm adopts a comprehensive measurement method, which combines the appearance and motion information of the target through linear weighting, and then enhances the weight of appearance features. The purpose is to improve the matching between recognition and tracking targets, thereby enhancing the algorithm performance. The I_DeepSORT algorithm includes ReID enhancement, trajectory processing, etc.

Firstly, in network structure, the improved deep CNN network structure information in the I_DeepSORT is shown in Table 1. In Table 1, the main content of this improvement is to increase the four-layer residual network. By increasing the depth of the model, the model can learn more layers and more complex feature representations, thus improving the ability to identify pedestrian identity, deepening the depth of the network and improving the identification accuracy. However, the network hierarchy deepening tends to slow down the network convergence speed, because the input distribution of the activation function may gradually approach the saturation region of the nonlinear function, resulting in the gradient disappearance of the lower neurons during back propagation. Batch normalization (BN) amplifies gradients and accelerates the learning rate of the network by re-normalizing the data with distribution shifts, which helps the response of the activation function to be more in the sensitive region of the nonlinear function. Therefore, after each residual network adds layers, BN operations are introduced in this section to ensure fast convergence even as the network depth increases.

**Table 1:** Optimized I_DeepSORT deep CNN structure.

Referred to as	Layer type	Step	Convolutional kernel size
Conv1	Convolution	1	3, 3
Conv2	Convolution	1	3, 3
Max_P3	Max pool	2	3, 3
Residual4	Residual	1	3, 3
BN	Batch normalization	–	–
Residual5	Residual	1	3, 3
BN	Batch normalization	–	–
Residual6	Residual	2	3, 3
BN	Batch normalization	–	–
Residual7	Residual	1	3, 3
BN	Batch normalization	–	–
Residual8	Residual	2	3, 3
BN	Batch normalization	–	–
Residual9	Residual	1	3, 3
BN	Batch normalization	–	–
Residual10	Residual	2	3, 3
BN	Batch normalization	–	–
Residual11	Residual	1	3, 3
BN	Batch normalization	–	–
Residual12	Residual	2	3, 3
BN	Batch normalization	–	–
Residual13	Residual	–	3, 3
BN	Batch normalization	–	–
Dense14	Dense connection	–	3, 3
Normalization	Two norm normalization	–	3, 3

Then the loss function of the network is redesigned. Common neural network loss functions include "0–1", Hinge, cross-entropy, and logarithmic, among which the latter two are the most widely used. Their calculations are shown in Eqs. (6) and (7), respectively.

6 $$L_{CE} = - \frac{1}{n}\sum\limits_{x} {\left[ {y\ln a + \left( {1 - y} \right)\ln \left( {1 - a} \right)} \right]}$$

In Eq. (6), $L_{CE}$ is the cross-entropy loss function of the binary classification problem. $y$ is the true label of the sample $x$. $a$ is the prediction output. $n$ is the total number of samples.

7 $$L_{\log } \left( {y,p\left( {y\left| x \right.} \right)} \right) = - \log p\left( {y\left| x \right.} \right)$$

In Eq. (7), $L_{\log }$ is the value of the logarithmic loss function. $p\left( {y\left| x \right.} \right)$ is the probability $y$ that the predicted outcome will be labeled given the input $x$. However, to minimize the inter-class error and maximize the intra-class difference, the Cosine softmax loss function trains the network, and the corresponding loss expression is shown in Eq. (8).

8 $$L_{CS} = - \sum\limits_{i = 1}^{N} {\sum\limits_{k = 1}^{C} {I_{{y_{i} = k}} \cdot \log p_{CS} \left( {y_{i} = k|r_{i} } \right)} }$$

In Eq. (8), $I_{{y_{i} = k}}$ is the indicator function, evaluated as 1. $p_{CS} \left( \cdot \right)$ is the Cosine softmax classifier in Eq. (9).

9 $$p\left( {y = k|r} \right) = \frac{{\exp \left( {\kappa \cdot \tilde{w}_{k}^{T} r} \right)}}{{\sum\nolimits_{n = 1}^{C} {\left( {\kappa \cdot \tilde{w}_{k}^{T} r} \right)} }}$$

In Eq. (9), $\kappa$ is the free scaling factor. $\tilde{w}_{k}^{T}$ is the normalized and inverted weight coefficient vector. $r$ is the basic representation corresponding to the features of the parameterized encoder network trained simultaneously as the classifier. $C$ is the maximum number of dataset labels. By minimizing the cross-entropy between the true label distribution and the Cosine softmax estimated probability, this loss function makes the estimated probability of the correct class close to 1 and the estimated probability of the rest of the classes close to 0.

Then, the trajectory processing and state estimation process of the I_DeepSORT algorithm are designed. The trajectory state can be represented in an 8-dimensional space $\left( {u,v,\gamma ,h,\dot{x},\dot{y},\dot{\gamma },\dot{h}} \right)$, which includes the center coordinates $\left( {u,v} \right)$ of the predicted bounding box, the predicted height $h$ and aspect ratio $\gamma$ of the pedestrian target box. The remaining four dimensions represent the velocity information of the above parameters relative to the image coordinates. With the help of a standard Kalman filter, the target motion prediction is carried out based on a uniform motion and linear observation model. The prediction result $\left( {u,v,\gamma ,h} \right)$ can be obtained by the algorithm, where the frame rate is standard 25 fps. The algorithm needs to set a counter for each target tracked, with the aim of counting during the Kalman filter prediction. The tracker's counter is reset when a trace is successfully matched to a detection. If the tracker consistently does not match the results within a specific time period, the tracker is removed from the tracker list. When a new finding appears (i.e., a finding that doesn't match an existing tracking list), a new tracker is created for it. If the location prediction of the newly tracked target matches the detection result in three consecutive frames, the algorithm considers the new target to appear. Otherwise, it is considered a "false alarm" and removes the tracker from the tracker list.

The information association and cascade matching rules in the algorithm are then designed. In terms of information association, the algorithm uses the Mahalanobis distance to describe the distance between the detection frame and the prediction frame, which evaluates the correlation of target motion information in Eq. (10).

10 $$L_{m}^{\left( 1 \right)} \left( {i,j} \right) = \left( {l_{j} - p_{i} } \right)^{T} X_{i}^{ - 1} \left( {l_{j} - p} \right)$$

In Eq. (10), $L_{m}^{\left( 1 \right)} \left( {i,j} \right)$ is the Mahalanobis distance between the detection frame and the prediction frame. $p_{i}$ is the predicted position of the $i$ tracker. $l_{j}$ is the predicted position of the $j$ detection target box. $X_{i}^{{}}$ is the matrix of covariance between the detected and averaged tracking locations. Taking into account the continuity of the target's motion state, the Mahalanobis distance matching filter is used, and the threshold is set to the 95% of the chi-square distribution. At the same time, considering that the correlation method of Mahalanobis distance is invalid when the camera is in motion, the target appearance information correlation is proposed. The calculation process is as follows.

Firstly, the eigenvectors $rr_{j}$ of each item $l_{j}$ ($\left\| {rr_{j} } \right\| = 1$) is calculated. The second step is to generate a relevant gallery for each tracking target, which contains the eigenvectors that are successfully associated with the first 100 frames. The third step is to calculate the minimum cosine distance $\left\| {rr_{j} } \right\| = 1$ between the gallery and the current frame detection result for each tracker. If the distance is less than the threshold, the association is successful. This distance is calculated in Eq. (11).

11 $$L_{{}}^{\left( 2 \right)} \left( {i,j} \right) = min\left( {1 - rr_{j}^{T} \cdot rr_{k} \left| {r_{k}^{\left( i \right)} \in R_{i} } \right.} \right)$$

In addition, in the final measurement, the improved algorithm uses a fusion measurement method to connect the target appearance and motion information through linear weighting to perform correlation measurements, so as to improve the matching degree between detection and tracking trajectories. This process can be described in Eq. (12).

12 $$c_{i,j} = \lambda l_{m}^{\left( 1 \right)} \left( {i,j} \right) + \left( {1 - \lambda } \right)l^{(2)} \left( {i,j} \right)$$

**Figure 4:**
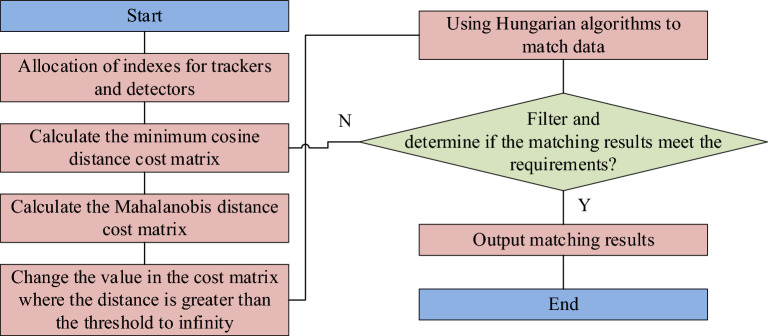
Cascade matching process.

In terms of cascade matching, if an object is occluded, the uncertainty of the Kalman filter prediction will increase with the increase of the observability of the state space. However, if the matching right of the same detection result is preempted by two trackers, the covariance of the tracking line that updates the location information will become larger due to the obscuration, resulting in an increase in the uncertainty of the tracking prediction position. In terms of the trajectory correlation of the detection results, it is easier to associate with objects with a long occlusion time, which greatly destroys the continuity of tracking. Cascade matching effectively solves this problem by matching trajectories with the same vanishing time to ensure that the closest object is given the highest priority. The specific flow of this idea is shown in Fig. 4.

**Figure 5:**
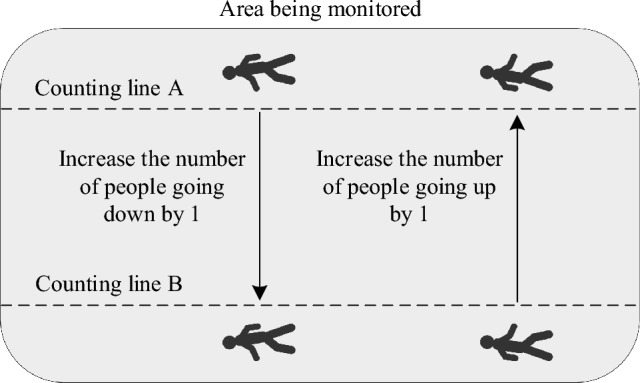
Flow counting rules.

At this point, the improved I_DeepSORT algorithm has been designed. Next, the technical rules of the crowd flow are determined, and the statistical model of the crowd flow is constructed by combining the improved I_YOLOv3 algorithm and the I_DeepSORT algorithm. In terms of crowd flow statistics rules, the virtual line setting is used in the model to count the crowd flow, and judge whether the pedestrian trajectory (formed by the center point of the tracking window) crosses the line to count the flow of people. Due to the high randomness of pedestrian behavior, especially considering the uncertainty of pedestrians' motion direction and detention situation, a two-way counting rule as shown in Fig. 5 is designed. As shown in Fig. 5, in the counting rule, depending on the pedestrian trajectory from A to B, it is specified as downward. If crossing line B, the number of people going down is added by 1. When the pedestrian crosses line A, the number of people going up is added by 1. The sum of the number of people going up and down is the total number.

**Figure 6:**
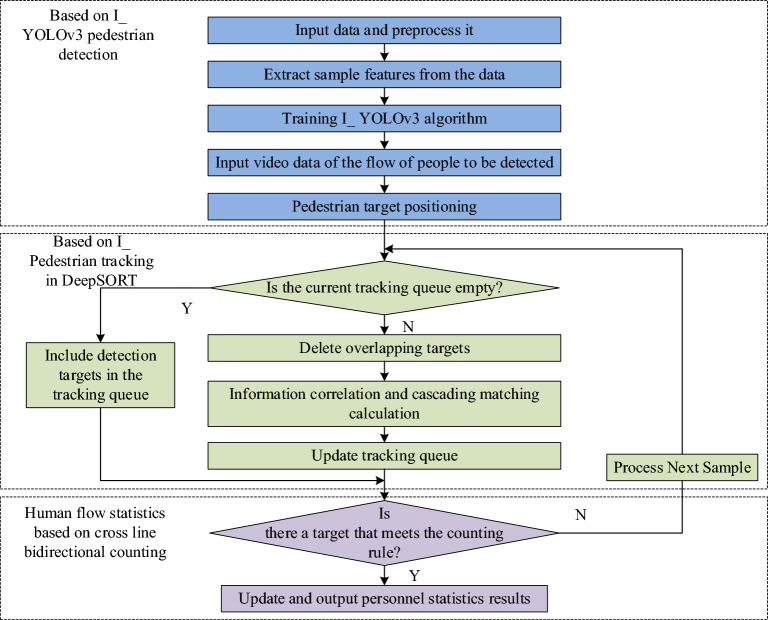
Statistical model of people flow recognition based on improved I_YOLOv3 and I_DeepSORT algorithms.

The improved I_YOLOv3 algorithm and I_DeepSORT algorithm are used for pedestrian target detection and pedestrian target tracking, respectively. A smart human flow statistical model is formed, as shown in Fig. 6. The model counts the number of people through the cross-line counting method, so as to achieve two-way people flow statistics.

## Performance test based on improved YOLO flow statistics model

After the statistical model for pedestrian flow recognition based on improved I_YOLOv3 and I_DeepSORT algorithm is designed, the experiment is carried out to verify the application value of the model in people flow statistics. The advantages and disadvantages of the model are discussed.

### Test plan construction

Testing experiments are conducted using two publicly available pedestrian datasets, Caletech and CUHK Occusion Pedstrian. Caltech dataset is widely used in the computer vision field, which contains multiple sub-datasets, such as Caltech101 and Caltech256. The CUHK dataset is a general term for several datasets published by the Chinese University of Hong Kong (CUHK). This dataset is the first large-scale pedestrian re-identification dataset sufficient for deep learning. 43,264 images of 13,264 individuals are collected from six existing personnel re-identification datasets (including CUHK03)^31^. Test experiments uses two publicly available pedestrian datasets, Huay Technology and CUHK occupy pedals. The team selects images with missing labels and completely blocking pedestrians, obtaining a total of 4268 valid data images. The IoU threshold of 0.3 is used for NMS, which allows the model to have a certain degree of fault tolerance when locating pedestrians. The appearance feature vector dimension 512 ensures the richness of feature expression, which helps improve the accuracy of detection and ReID. Weight attenuation of 0.0005 is used for regularization to prevent over-fitting and ensure the model generalization ability on the training set. The dataset partitioning of 7:3 ensures sufficient data for model training while retaining sufficient test data to evaluate model performance. The research team screens out the images with missing labels and completely occluded pedestrians, obtaining a total of 4268 valid data images. The dataset includes a testing set and a training set at 7:3.

Accuracy personAP, Log Average Miss rate (LAMR), Multi Object Tracking Accuracy (MOTA), and Multi Object Tracking Accuracy (MOTP) are used as evaluation metrics for model performance in the test. PersonAP is calculated in Eq. (13).

13 $$personAP = \Sigma precision_{C} /N\left( {Tot\_Ima} \right)_{C}$$

In Eq. (13), $\Sigma precision_{C}$ is the sum of the average recognition precision of all pedestrian types in all images. $N\left( {Tot\_Ima} \right)_{C}$ is the number of images containing pedestrian objects in all tested images.

The LAMR calculates the average false detection rate over the interval [10^–2^,100] and takes a logarithmic average of 9 points uniformly in Eq. (14).

14 $$LAMR = exp\left( {1/9 \cdot \sum\limits_{i = 1}^{9} {\ln \left[ {missrate\left( {10^{ - 2.25 + 0.25i} } \right)} \right]} } \right)$$

In Eq. (14), $missrate\left( \cdot \right)$ is the recognition error rate. LAMR is the ratio of the number of images with recognition errors to the total number of images tested. Other conditions remain constant, the smaller the LAMR value, the higher the detection accuracy of the model.

The MOTA calculation method is shown in Eq. (15).

15 $$MOTA = 1 - \left( {FN + FP + ID\_SW} \right)/GT$$

In Eq. (15), $FN$, $FP$, $ID\_SW$, and $GT$ represent the number of false negative cases, the number of false positive cases, the number of identity switches, and the number of ground truth objects, respectively.

The MOTP calculation method is shown in Eq. (16).

16 $$MOTP = \frac{{\sum\nolimits_{t,i} {d_{t,i} } }}{{\sum\nolimits_{t} {c_{t} } }}$$

In Eq. (16), $i$ represents the detection target. $d_{t,i}$ represents the average measurement distance between the detection target $i$ and its true value in all frames. $c_{t}$ represents the number of successful matches.

Table 2 shows the parameter setting results of the software environment, hardware environment, and improved flow recognition model in the test. Model design and hyper-parameter comparison are determined by manual experience to determine the range, and then debugged based on the grid parameter method.

**Table 2:** Software and hardware environment, improved flow identification model parameter setting scheme.

Type	Item	Number	Set results
Software environment	Operating system	01	Windows 10 professional edition
	Programming language	02	Python 3.0
	Algorithm toolkit	03	Tensorflow
Hardware environment	Central processing unit	11	i7-8600
	Running memory size	12	16GB
	Graphics processor	13	GeForce GTX 1070
Argument	Learning rate	21	0.002
	Training mode	22	Graphics processing unit
	Sample size for single batch training	23	128
	Epochs	24	490
	Does the hidden layer have an offset term	25	And
	Parameter initialization method	26	Random initialization

In order to improve the reliability of the test results, the common target detection algorithms YOLOv3, CenterNet proposed by^32^, and the accepted field block network (RFBNet) proposed by^33^ are used as comparison models.

### Analysis of test calculation results

Firstly, in order to verify the superiority of the proposed method, the algorithm ablation experiment is conducted, and compared with the newly proposed CenterNet algorithm and RFBNet algorithm. The average accuracy of the target detection algorithm is evaluated by Mean Average Precision (mAP), and the accuracy of multi-target tracking is evaluated by MOTA. MOTP is used to evaluate the accuracy of the tracking results, and Frames Per Second (FPS) is used to evaluate the real-time performance of the algorithm. Model size is sued to evaluate the size and storage requirements of the model. The experimental results are shown in Table 3.

**Table 3:** Results of the algorithm ablation experiments.

Experiment group	mAP (%)	MOTA (%	MOTP (cm)	FPS	Model size (MB)
Baseline	75	60	300	20	200
I_YOLOv3	77	61	280	25	180
_DeepSORT(No ResNet)	76	62	290	23	190
_DeepSORT(ResNet-4)	78	65	270	22	210
CenterNet	80	66	260	30	220
RFBNet	82	67	250	28	230

**Figure 7:**
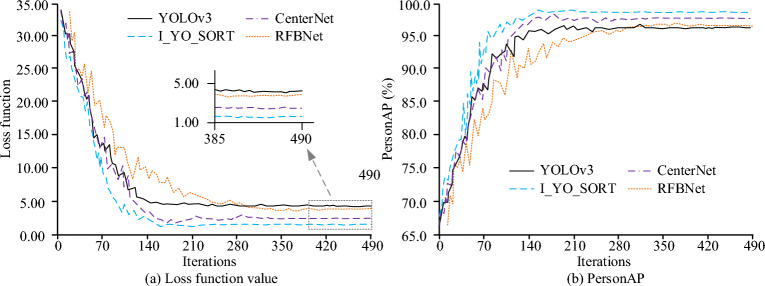
Comparison of training effects of pedestrian flow statistical models.

According to the experimental results in Table 3, the algorithm proposed in this study achieved significant improvements in multiple aspects. In terms of object detection, I_YOLOv3 improved mAP by 2% compared with Baseline, demonstrating better detection accuracy. In the DeepSORT algorithm with four layers of ResNet added, MOTA increased by 5% and MOTP decreased by 30 cm, indicating a significant improvement in the multi-target tracking accuracy. Although the tracking accuracy slightly decreased, it was still within an acceptable range. In addition, the FPS of I_YOLOv3 increased from 20 to 25, resulting in improved real-time performance. At the same time, the model size reduced by 20MB, indicating that the algorithm was optimized in terms of computational efficiency and storage requirements. Compared with CenterNet and RFBNet, although there are slight shortcomings in mAP and MOTA, I_YOLOv3 and its combination with DeepSORT perform excellently in overall performance, especially in real-time performance and model size, verifying the superiority of the proposed method. The performance of each model in the training process is analyzed, as shown in Fig. 7. The "I_YO_SORT" represents the statistical model designed in this study. The horizontal axis represents the number of iterations, and the vertical axis in Fig. 7a,b show the loss function value and the PersonAP value, respectively. As iterations increased, the loss function of each statistical model gradually has decreased, and the PersonAP value gradually has increased and tended to be stable. I_YO_SORT model completed convergence when training times were 168, and the convergence speed was faster. After 300 iterations, the training of each model was completed, and the loss functions and PersonAP values of the I_YO_SORT, CenterNet, RFBNet, and YOLOv3 models were 0.86, 1.47, 4.82, and 4.93, and 98.7%, 96.8%, 95.3%, and 94.9%, respectively.

**Figure 8:**
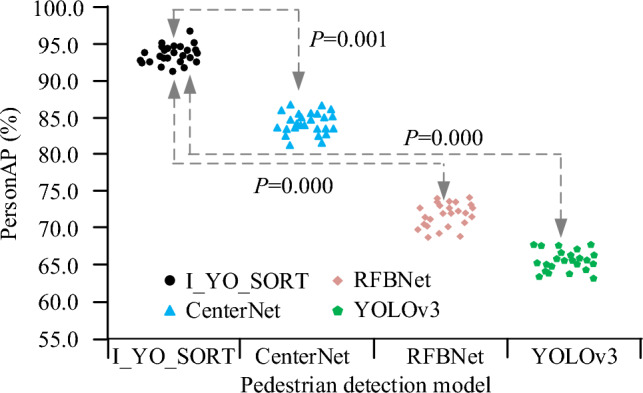
Comparison of PersonAP and detection time in the testing set of the flow statistical model.

After training each model, the study conducts application experiments in many crowded places in Shanghai. The statistical results of pedestrian detection PersonAP and detection time are shown in Fig. 8. The horizontal and vertical axes describe the values of different pedestrian detection models and PersonAP indicators. To test the model stability, each test scheme is repeated 30 times. The difference is tested by T-test, and the significance level of the difference is 0.05. Figure 8 showed that the median PersonAP of the I_YO_SORT, CenterNet, RFBNet, and YOLOv3 pedestrian detection models were 94.2%, 84.5%, 71.3%, and 65.1%, respectively, and the *P* values of the T-test for last three models and the I_YO_SORT model were all less than 0.05, which was considered to be significant.

**Figure 9:**
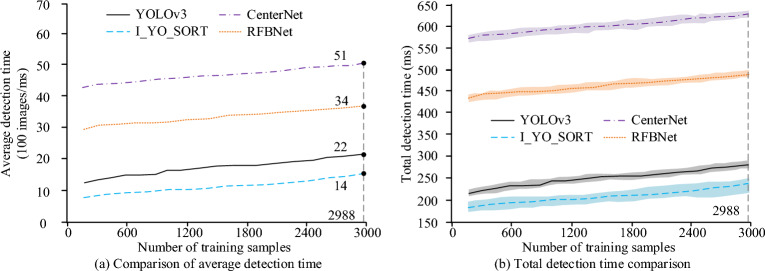
Testing set detection time of each model under different number of training samples.

To test the running speed and sensitivity of each person detection model to the number of training samples, the testing set detection time of each model under different training sample numbers is tested, as shown in Fig. 9. Each protocol is also performed in 30 replicates. In Fig. 9a,b, the horizontal axis represents the number of samples participating in the model training, with the maximum value being 2988 images for the entire training set, and the vertical axis representing the average detection time of 100 images and the total detection time of the test set, respectively, (ms). The populated area in Fig. 9b represents the range of the maximum and minimum values of the total time spent on the corresponding model, which is used to describe the fluctuation of the total detection time. As the number of training samples increases, the average detection time and total detection time of each model generally show an upward trend, because the more samples participate in model training, the higher the overall complexity of the CNN after the model. Therefore, the connections between neurons become more complex, which increases the computational time used, but the increase is relatively small. When the training set was 2988 images, the total detection time of the I_YO_SORT, CenterNet, RFBNet, and YOLOv3 pedestrian detection models was 216 ms, 638 ms, 482 ms, and 271 ms, respectively.

**Figure 10:**
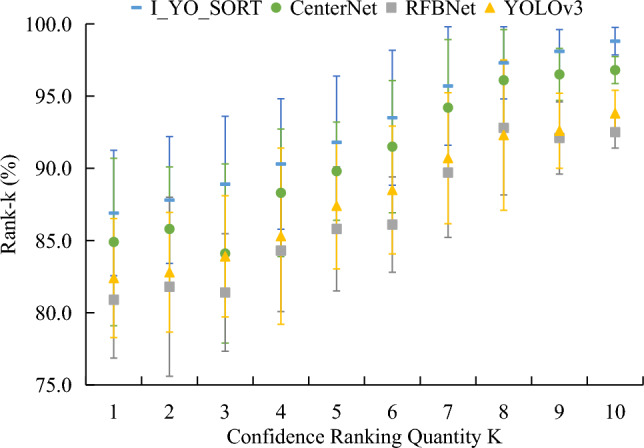
Comparison of Rank-k indicators of each model testing set.

The Rank-k metric evaluates each detection accuracy, which represents the probability that the top k images contain the correct label in the confidence level of the prediction output. The statistical results are shown in Fig. 10. The horizontal axis is the value of k, and the vertical axis is the corresponding Rank-k accuracy. As k increased, the Rank-k accuracy of each model also has increased. The average Rank-k accuracy for the I_YO_SORT was always higher than that of the other comparison models. When k was 1 and 10, the average Rank-k accuracy of I_YO_SORT, CenterNet, RFBNet, and YOLOv3 models was 87.2%, 84.8%, 82.6%, and 81.3%, and 98.6%, 96.2%, 92.5%, and 91.0%, respectively.

Table 4 shows the comparison of four multi-target tracking performance evaluation indexes: MOTA, MOTP, the proportion of hit trajectory assumptions to trajectory truth (Mostly Tracked, MT), and the proportion of lost target trajectory to trajectory truth (Mostly Lost, ML) of each model under different Rank-k conditions. From Table 4, the values of the I_YO_SORT model on MOTA and MOTP were higher than those of the other three models under the same conditions, indicating that the former had higher multi-target tracking accuracy. At the same time, the MT and ML indicators of the I_YO_SORT model were lower than those of the other three models, indicating that the former improved the integrity of the overall tracking trajectory.

**Table 4:** Comparison of multi target tracking performance indexes of various models under different Rank-k conditions.

Model name	Towards	TYPE (%)	MOTP (%)	MT (%)	ML (%)
I_YO_SORT	1	76.8	82.5	29.2	37.2
	5	93.6	96.2	26.3	33.6
	10	98.5	98.3	22.5	25.2
CenterNet	1	72.1	78.1	36.2	49.3
	5	90.5	91.5	32.6	45.1
	10	94.2	93.2	29.4	40.8
RFBNet	1	64.0	72.4	42.5	53.6
	5	84.6	87.9	36.2	48.5
	10	88.7	89.6	31.4	46.2
YOLOv3	1	67.4	70.3	53.6	51.3
	5	87.2	84.2	43.6	47.2
	10	91.3	86.5	38.5	43.6

**Figure 11:**
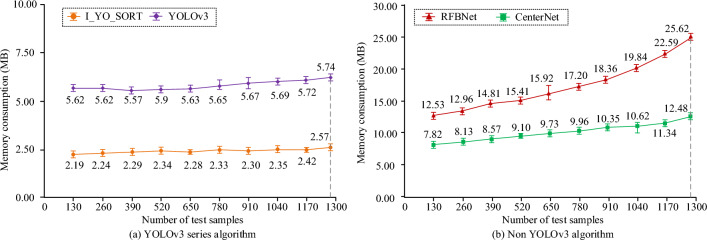
Memory consumption for each model testing set tracking counts.

Figure 11 compares the memory consumption of each model in the tracking count of the testing set. In Fig. 11, the horizontal axis represents the number of testing set samples participating in the calculation, and the vertical axis represents the corresponding memory consumption. The gray dotted line in Fig. 11 corresponds to the horizontal axis value, which is the 1280 images of the entire test set. From Fig. 11, as the number of samples increased, the memory consumption of the I_YO_SORT and YOLOv3 models has changed slightly. The memory consumption of the CenterNet model increased, and the RFBNet increased the fastest. When the samples participating were 130 and 1280, respectively, the memory consumption of the I_YO_SORT, CenterNet, RFBNet, and YOLOv3 models was 2.42 MB, 7.81 MB, 12.85 MB, 5.31 MB, and 2.57 MB, 12.48 MB, 25.62 MB, and 5.74 MB, respectively.

**Figure 12:**
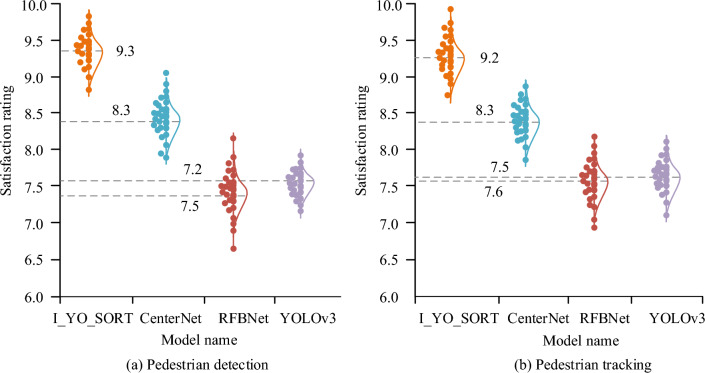
Comparison of subjective evaluation scores.

From the perspective of subjective evaluation, the statistical effect of crowd recognition of each model is analyzed. 20 domestic object detection experts are invited to participate in the evaluation, and they are required to conduct multi-dimensional satisfaction scores on the flow statistics of each model. The score is 10 points. The higher the score, the higher the satisfaction, the evaluation results are shown in Fig. 12. The median subjective scores of pedestrian detection and trust tracking of I_YO_SORT, CenterNet, RFBNet, and YOLOv3 models were 9.3, 8.3, 7.2, 7.5, and 9.2, 8.3, 7.5, respectively. 7.6. The overall score of the I_YO_SORT was higher than that of the comparison model.

## Conclusion

To solve the insufficient recognition accuracy and high resource consumption, a lightweight intelligent statistical model for people flow based on improved YOLOv3 and ReID was designed. The test results are as follows. I_YO_SORT model completed convergence when the number of training times was about 168 times, and the convergence speed was faster. The median PersonAP of the I_YO_SORT, CenterNet, RFBNet, and YOLOv3 pedestrian detection models were 94.2%, 84.5%, 71.3%, and 65.1%, respectively, and the *P* values of the T-test for last three models and the I_YO_SORT model were all less than 0.05, which was considered to be significantly different. When the training set was 2988 images, the total detection time of the I_YO_SORT, CenterNet, RFBNet, and YOLOv3 pedestrian detection models was 216 ms, 638 ms, 482 ms, and 271 ms, respectively. With the increase of k, the Rank-k accuracy of each model also increased, but the average Rank-k accuracy of the I_YO_SORT was always higher than that of the other comparison models. The values of the I_YO_SORT model on MOTA and MOTP were higher than those of the other three models under the same conditions. Meanwhile, the MT and ML indicators of the I_YO_SORT model were lower than those of the other three models. When the number of samples participating in the test was 130 and 1280, respectively, the memory consumption of the I_YO_SORT, CenterNet, RFBNet, and YOLOv3 models was 2.42 MB, 7.81 MB, 12.85 MB, 5.31 MB, and 2.57 MB, 12.48 MB, 25.62 MB, and 5.74 MB, respectively. The median subjective scores of pedestrian detection and trust tracking of the I_YO_SORT, CenterNet, RFBNet, and YOLOv3 models were 9.3, 8.3, 7.2, and 7.5, and 9.2, 8.3, 7.5, and 7.6, respectively, and the overall scores of the I_YO_SORT were higher. The test results show that the flow statistics model designed in this study has higher tracking accuracy, faster statistical speed, and less resource consumption. Although the improved intelligent human traffic statistics model based on YOLOv3 and ReID designed in this study has significantly improved performance, there are still some research shortcomings. Firstly, the model has not yet been widely deployed and tested in actual commercial grade products. Secondly, in complex and constantly changing environments, the performance of the model may be affected. To make up for these shortcomings, it is necessary to continue exploring the performance optimization of model under extreme weather conditions in the future, and integrating new technologies into existing models to continuously improve its performance. Through these efforts, it is expected to provide more accurate, efficient, and intelligent solutions for crowd management in public places.

## Data Availability

All data generated or analyzed during this study are included in this published article.
